# Disease- and Drug-Related Knowledge Extraction for Health Management from Online Health Communities Based on BERT-BiGRU-ATT

**DOI:** 10.3390/ijerph192416590

**Published:** 2022-12-09

**Authors:** Yanli Zhang, Xinmiao Li, Yu Yang, Tao Wang

**Affiliations:** 1 College of Business Administration, Henan Finance University, Zhengzhou 451464, China; 2 Business School, Henan University, Kaifeng 475004, China; 3 School of Information Management and Engineering, Shanghai University of Finance and Economics, Shanghai 200433, China; 4 China Banking and Insurance Regulatory Commission Neimengu Office, Hohhot 010019, China

**Keywords:** online post, online health communities, knowledge recovery, relationship extraction, deep learning, disease medication, health management

## Abstract

Knowledge extraction from rich text in online health communities can supplement and improve the existing knowledge base, supporting evidence-based medicine and clinical decision making. The extracted time series health management data of users can help users with similar conditions when managing their health. By annotating four relationships, this study constructed a deep learning model, BERT-BiGRU-ATT, to extract disease–medication relationships. A Chinese-pretrained BERT model was used to generate word embeddings for the question-and-answer data from online health communities in China. In addition, the bidirectional gated recurrent unit, combined with an attention mechanism, was employed to capture sequence context features and then to classify text related to diseases and drugs using a softmax classifier and to obtain the time series data provided by users. By using various word embedding training experiments and comparisons with classical models, the superiority of our model in relation to extraction was verified. Based on the knowledge extraction, the evolution of a user’s disease progression was analyzed according to the time series data provided by users to further analyze the evolution of the user’s disease progression. BERT word embedding, GRU, and attention mechanisms in our research play major roles in knowledge extraction. The knowledge extraction results obtained are expected to supplement and improve the existing knowledge base, assist doctors’ diagnosis, and help users with dynamic lifecycle health management, such as user disease treatment management. In future studies, a co-reference resolution can be introduced to further improve the effect of extracting the relationships among diseases, drugs, and drug effects.

## 1. Introduction

The incidence of chronic diseases is rapidly increasing worldwide [1]. Chronic diseases represented by hypertension, stroke, diabetes, and coronary heart disease seriously endanger human health and become important diseases threatening people’s health. According to the World Health Organization, users with diabetes, hypertension, and cardiovascular problems rely on functional medications every day [1]. Therefore, patients and doctors must be aware of the effects of commonly used drugs [2]. Disease management is a crucial aspect of health management [3]. Determining drug effects is the primary concern of drug management [4]. The phrase “diseases, drugs, and drug effects (DDEs)” refers to an obvious improvement effect or other effects observed when drugs are used to treat diseases in different individuals [5]. Moreover, DDEs can reflect the effects experienced by different individuals after taking medicine. Consequently, a patient can consult the physician regarding continuing, stopping, replacing, or intervening the treatment scheme depending on the drug’s effects. In general, drug effects corresponding to various diseases are obtained via clinical diagnoses; however, these effects vary for different people, and those identified in limited clinical cases are far from sufficient. Online health communities (OHCs) provide a convenient and fast communication channel, and after taking medication, users can review symptoms and drug effects in OHCs [6]. For example, users can understand the drug effects experienced by others over time; this enables a better understanding of the drug effects for various diseases with respect to users with similar conditions [2,7,8] and avoid adverse effects [9,10,11]. Although this user-generated content (UGC) in OHCs is timely and effective, a large amount of UGC remains unused for disease management.

Scholars mostly studied the extraction of the relationship among diseases, symptoms, and tests [12], and the relationship between diseases, drugs, and efficacy garnered the attention of many scholars [13,14,15]. Extracting the relationship among DDEs from the UGC of OHCs can generate a large amount of information regarding disease medication, which can be used to establish, supplement, and improve the existing knowledge base of DDEs and can assist clinical decision making [15]. Knowledge extraction results are also a key step in establishing a medical knowledge map [16]. At the same time, the drug effects shared by these large user groups provide support for evidence-based medicine [17]. However, research with respect to extracting the relationship among DDEs from unstructured texts in OHCs and then establishing a knowledge base of disease medication effects based on the UGC in OHCs is scarce.

Studies on information extraction in the field of biomedical and health informatics are mostly based on biomedical literature summaries [18], hospital discharge summaries [19], and electronic medical records [20]. The words and sentences in these corpora are relatively structured. However, extracting relationships from OHCs that contain colloquial and poorly structured questions and answers (Q&As) is difficult. Therefore, this study aims to extract the relationship among DDEs from doctor–patient Q&A data in OHCs, and it focuses on the relationship extraction of DDEs as an information extraction problem. Considering the popularity of bidirectional word embedding encodings and conversion technology and in order to better obtain the semantic context, our research utilizes BERT word embedding technology, deep learning technology, and the attention mechanism (ATT), thereby better representing semantic relations. We construct a BERT-BiGRU-ATT network model based on ATT, which is devoted to the problem of extracting relationships among DDEs from OHCs. In addition, time-series data based on a user’s disease medication are obtained via relation extraction to show the disease treatment process, which provides a strong guarantee for dynamic health management based on the life cycle.

This paper is committed to making the following three contributions to the literature: first, our study extends the research on DDEs knowledge extraction to OHCs and expands the data sources of disease–drug research; second, our research expands the research direction of knowledge extraction from OHCs; third, based on the results of the research, we provide a better understanding of DDE’s knowledge extraction.

The article is organized as follows. We describe how our work relates to existing literature streams in Section 2 and provide the research Methodology in Section 3. The experimental setup is presented in Section 4. Experimental Results are provided in Section 5. We then present Discussion and Conclusions, the implications of our findings and a discussion of the limitations in Section 6.

## 2. Related Works

### 2.1. Research on Knowledge Extraction from OHCs

There are several users in OHCs, and user-generated content is now rich enough for knowledge extraction. For example, the MedDRA was used to extract the knowledge of drugs and drug effects from the Spanish drug effect database [15]. There is also a rule-based approach for extracting knowledge on dietary recommendations from open data [21]. In addition, disease- and drug-related knowledge extraction in the online health community plays a significant role in the field of relationship extraction. For example, adverse drug events (ADEs) were extracted from the user-generated content of OHCs [8,9,10], and new indications for drugs outside the drug label were found from online communities [2,7].

### 2.2. Research on Medical Text Knowledge Extraction

Initially, scholars mostly used pattern matching and machine learning methods to extract knowledge related to diseases and drugs from medical texts. The former adopted syntactic structure analyses, and relation extraction is carried out by expert-defined rules. These methods generally have a low recall rate. Iqbal et al. extracted the relationship between drugs and side effects from electronic medical records based on rules [20]. In the I2B2/VA challenge task, the relationship between medical concepts in patients’ clinical records was extracted; specifically, the following three types of medical relationships were extracted using machine learning [22]. In addition, support vector machine and kernel function methods are widely used for relationship extractions in the biomedical field. This study uses the abundant features of support vector machines to extract knowledge between chemical substances and diseases from research articles in PubMed [23]. Furthermore, it employs multiple algorithms from machine learning to extract knowledge between cures, preventions, and side effects from clinical records [24] and to extract relationships related to patients’ medical problems (disease, examination, and treatment) from discharge summaries [19]. Machine learning has been widely used in the knowledge extraction of adverse drug events (ADEs) [9,10,11], new indications for on-label drug use [2], drug–drug interactions [14], and the relationship between chemical substances and disease [23]. Most of the relational extraction corpora of the above studies come from relatively structured texts (for instance, electronic medical records). However, there are few studies that have extracted the relationship of DDEs from a large number of colloquial and unstructured texts.

### 2.3. Research on Medical Knowledge Extraction Based on Deep Learning

Artificial intelligence has been extensively used in the industry and in medical practice. The methods of machine learning require specific domain knowledge and artificial features in relation extraction tasks, which require considerable manpower. Deep learning experienced a period of development and influenced many fields and industries [25]. It is a new type of artificial intelligence technology. In various information extraction tasks, it uses multi-layer cross-connected nodes to establish a multi-relation classification model for input data, which can automatically and intelligently extract features, thus saving a tremendous amount of manual work. Deep learning has been widely used when processing health information. The research used the GRU model to extract knowledge on bacteria-related information from the academic literature of biomedicine [26]. Luo et al. achieved good results in evaluating the relationship among multiple medical problems on the i2b2/VA relationship classification challenge dataset [27]. Yadav et al. used an efficient multi-task deep learning framework to classify the relationship between drug–drug interactions, protein–protein interactions, and medical concepts [28]. Deep learning is also widely used in medical practice, such as nodule detection [29], medical labeling, and scanning [30]. Moreover, it is widely used in the knowledge extraction of adverse drug events (ADEs) [13], drug–drug interactions [31], and the therapeutic effect of drugs on diseases [32,33,34]. Health informatics and natural language processing have become important fields with respect to deep learning technology application [35]. Word correlation training is used to convert words into word vector representation, and BERT word vector and word translation representation are important technologies for assisting deep learning.

However, the current research on extracting medical knowledge primarily focuses on relatively structured small sample data, such as medical literature summaries. However, these corpora are relatively small in scale, and relatively limited knowledge was obtained by relation extraction. In addition, the application of these research methods on a large sample corpus of colloquial and unstructured texts is not ideal. There is little research on how to apply the technology of deep learning to large oral and unstructured sample data to extract the relationships among DDEs, but billions of users in OHCs have generated exceptional amounts of data. By using these large amounts of data, we can obtain valuable knowledge that can help improve the existing knowledge base, thereby enabling auxiliary clinical decision support. Because deep learning is an important development in the field of text knowledge extraction, it is a crucial method for achieving the DDE relationships extraction, which is an objective of this study.

## 3. Methodology

**Figure 1:**
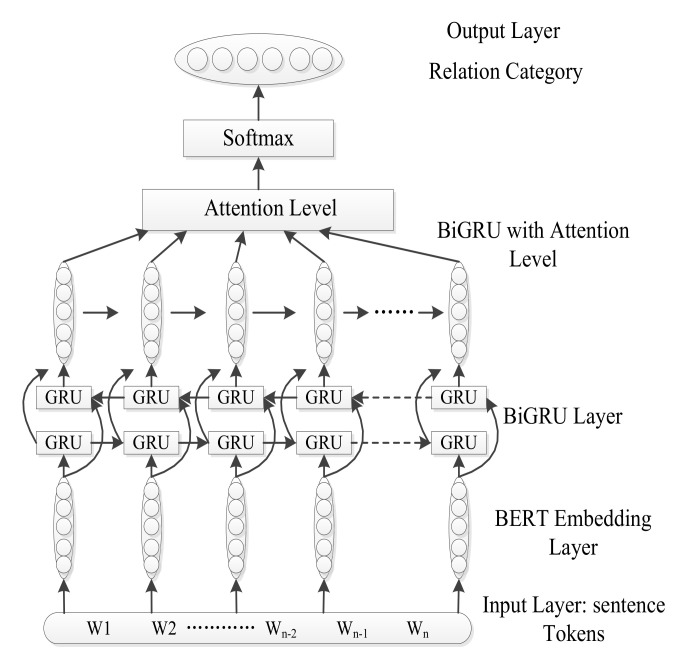
BERT-BiGRU-ATT.

The proposed knowledge extraction model of BERT-BiGRU-ATT is shown in Figure 1. The methods of BERT-BiGRU-ATT are described as follows: Input sentences are converted to BERT word embeddings through a bidirectional GRU layer and by a weighted distribution of attention mechanisms. Then, the relationship between two entities is classified by a softmax function. Finally, the model gains a relationship type related to the maximum probability.

### 3.1. Bert Word Embeddings

The early classical word embedding algorithm, word2vec, was proposed by Mikolov et al. [36] and is used for feedforward neural network training to predict the next word and then the preceding word of a given word. Recent research introduced a new algorithm for calculating word embeddings: BERT, which is a milestone in unsupervised training language models based on transformers [37,38,39]. The transformer is represented by the bidirectional encoder and decoder and uses a sequence-to-sequence model built using an attention mechanism. It can focus on different positions of the input sequence to calculate the representation ability of the sequence. BERT can overcome the limitation that word2vec has of only one static representation for a token and with no context. In addition, BERT overcomes the limitation of the single attention mechanism, wherein only the left or right context is combined. Because of the good effects of BERT in both theory and practice, this study used BERT to extract the relationship of DDEs to help provide better drug-related contexts.

### 3.2. BIGRU

Gated recurrent units (GRUs) [40] simplify the model of LSTM. Specifically, they merge the forgetting and inputting gates of LSTM into an update gate. GRU architecture can handle relationship classification tasks more efficiently. The update gate, z_t_, determines the extent to which the information from the previous state is forgotten and what information from the new content is to be added. The reset gate, r_t_, controls how the extent of the previous hidden state and the current input is ignored. The t-th update gate, reset gate, and cell state are calculated as follows:

z_t_ = σ(W_z_·[h_t−1_, x_t_]), (1)

r_t_ = σ(W_r_·[h_t−1_, x_t_]), (2)

(3) $$\tilde{\;h_{t}}=\tan h(W\cdot [r_{t-1}\times h_{t-1},\;x_{t}]),$$

(4) $$h_{t}=(1-z_{t})\;\times \;h_{t-1}+z_{t}\;\times \;\tilde{h_{t}},$$

where $\tilde{h_{t}}$ is the new memory content, which is obtained from h_t−1_, ht is the new cell state, and x is the current input.

### 3.3. Attention Mechanism

The influence of different input sequences on the output is different in semantic information. Specific words have an important influence on the output, whereas certain words are irrelevant. The attention mechanism was used to identify words that have a significant impact on the output, assigning it a higher weight such that its semantic information can be fully obtained [41].

Attention mechanism H = [h_1_,h_2_,…,h_T_] represents the matrix of the output vectors produced by the BiGRU layer, and T represents the sentence’s length. The eigenvector, r, of the sentence was obtained using the summation of the output vectors multiplied by the weight:

M = tanh(H),(5)

α = softmax(ω^T^M), (6)

γ = Hα^T^, (7)

where M denotes the state after the activation function, α denotes the obtained attention weight, and γ denotes the output vector after weight summation processes. $H\in R^{d^{\omega }\times T}$, d^ω^ is the word embedding, ω is the parameter vector after training, and the dimensions of ω, α, and γ are d^ω^, T, and d^ω^, respectively.

Finally, the sentence representation after the attention mechanism is obtained as follows.

h* = tanh(γ),(8)

### 3.4. Softmax Output Layer

The relationship’s extraction is considered a classification task. The sentence feature vector following the attention mechanism was classified by the softmax function classifier to output the probability of the predicated relationship type of the entity pairs.

(9) $$\hat{P}(y|S)=\mathrm{softmax}(W^{\left(s\right)}\;{h_{s}}^{*}+b^{\left(s\right)}),$$

(10) $$\hat{y}\;=\mathrm{argmax}\hat{P}(y|S),$$

To evaluate the experimental results, the prediction and annotated values were defined as 1 and the inconsistency was defined as 0. Furthermore, F (F-score), R (recall), and P (precision) were used to evaluate the results. For the overall performance of the model, the micro-average was used for evaluation, where TP denotes true positives, FP denotes false positives, and FN denotes false negatives. To evaluate the overall performance of the model, the microaverage was used.

(11) $$\mathrm{micro}\;P=\frac{\sum_{c}\mathrm{TP}\left(c\right)}{\sum_{c}\mathrm{TP}\left(c\right)+\sum_{c}\mathrm{FP}\left(c\right)},$$

(12) $$\mathrm{micro}\;R=\frac{\sum_{c}\mathrm{TP}\left(c\right)}{\sum_{c}\mathrm{TP}\left(c\right)+\sum_{c}\mathrm{FN}\left(c\right)},$$

(13) $$\mathrm{micro}\;F-\mathrm{score}=\frac{2*\mathrm{micro}\;P*\mathrm{micro}\;R}{\mathrm{micro}\;P+\mathrm{micro}\;R},$$

## 4. Experimental Setup

### 4.1. Data Description

The dataset in the knowledge extraction task is from the Ask and Answer website (www.120ask.com, abbreviated as 120ask, accessed on 1 June 2020), which is China’s most popular Q&A OHC. On this site, tens of thousands of real name-certified doctors with different clinical titles and working at different hospital levels, from different regions, provided free Q&A consulting services online. It contains a wealth of information, including the following aspects: Q&A data (problem and reply, reply subproblem/child, etc.) based on the interactions of doctors with the users, the personal information of the users (age, gender, and location) and the doctors (title, professional portrait, and hospital information), and user adoption labels. Therefore, this website is highly suitable for data extraction.

**Figure 2:**
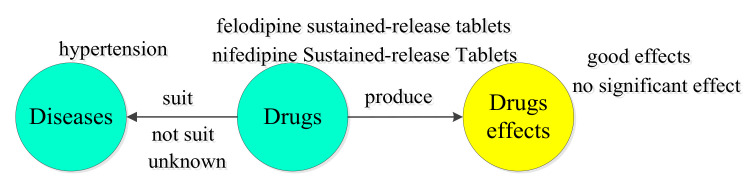
Diagram of the extracted relationships.

We used an automatic python crawler to download Q&A data from 120ask.com to test the research model of our knowledge extraction task. Data cleaning involved deleting users communicating in languages other than Chinese, empty questions or replies, and invalid disease information. In addition to the posts that were blocked because the issue contains sensitive words and violates community rules, we used the disease library and drug library to delete users who have never consulted about the disease and drug-related issues according to their user IDs. After data cleaning, 180 million Q&A data provided by 60 million users were preprocessed [42]. Considering cardiovascular disease as an example, following word segmentation and the execution of entity recognition tasks for the physician–patient Q&A corpus, four relationships were annotated for model training, testing, and predictions. We recruited two medical students to perform the data annotation task. The Cohen’s Kappa value with respect to the internal agreement measure reached 0.88. As shown in Figure 2, the four relationships are as follows: (1) drug-suit-disease (DsDIS); (2) drug-not-suit-disease (DnsDIS); (3) drug-produce-effect (DpEFF); and (4) others (i.e., no relationship between entities). For these entities, disease refers to an unhealthy state or doctor’s diagnosis result, and the drug effect refers to any observed changes in bodily functions after the drug is taken, thereby determining the therapeutic and pharmacological effects of the drug.

Cardiovascular diseases were considered examples of the training and testing processes from the 120ask Q&A data in OHC from 2015 to 2020. There were 532,486 cardiovascular disease Q&A records generated by 49,586 users in the 120ask Q&A dataset. The data from users who asked questions more than five times were extracted. Consequently, a total of 1927 users and 15,572 Q&A data were annotated in terms of 9732 relationships. All data were cleaned and preprocessed; according to the ratio of 3:1, we divided the training set and test set. Thereafter, the training data were input into the model for training, and the model’s parameters were obtained. Finally, the results on the test set were obtained. The annotation data are presented in Table 1.

### 4.2. Parameters Setup

The experiment used the open-source library Keras [43] based on TensorFlow [44] and the program language of Python3 to obtain knowledge extraction results. Word embeddings were obtained using the BERT model. Furthermore, the BRT word embedding used a classical Chinese pretraining model: BERT-wwm. In addition, for the flexible short text of the OHCs, the corpus training set of the Q&A OHCs was used for further pretraining. Finally, the proposed BERT-BiGRU-ATT sequence model attempted to obtain a classification probability by using model training and parameter tuning. These parameters were adjusted in the appropriate range, and the parameters that yielded the best results were as follows: word embedding at 150, batch size at 32, epoch at 20, hidden layer node number at 256, and dropout at 0.5 [45].

## 5. Experimental Results

### 5.1. Main Results

Using the BERT-BiGRU-ATT model, the knowledge extraction results are presented in Table 2. The overall F-score of DsDIS reached 88.40%. The F-score of DnsDIS reached 85.67%, and the F-score of DpEFF was 85.15% in the dataset. Because of the difference in the number of annotated relationship samples and the classified degree of difficulty of different relationships, the knowledge extraction results are significantly different.

To verify the validity and feasibility of the model, two groups of experiments were employed to verify the effectiveness of the DDE extraction model BERT-BiGRU-ATT proposed in this study. The first group of experiments compared the effects of the BERT pretraining model with other word embedding representations, such as word2vec, fastText [46], and GPT [47], on the Q&A OHC’s short text; the results are presented in Table 3. Another group of experiments compared the extraction effect of the BERT-BiGRU-ATT model in the DDE relationship with other excellent models of the LSTM, GRU, and BERT-GRU models; the results are presented in Table 4. All experiments used ten-fold cross-validation, training, and testing on the corpus.

The final results confirmed the superiority of BERT compared to other three-word embeddings in relationship extractions. Furthermore, in terms of the model’s effect, compared with those of the other three models, the BERT-BiGRU-ATT model exhibited better precision, recall, and F-score, reaching 88.46, 86.09, and 87.26%, respectively.

The results of the first experiment indicate that different word embedding extraction methods have different degrees of influence on the effect of the model. The FastText method is better than word2vec because it considers the subwords when training word embeddings, thereby introducing character-level n-grams to better address long words and low-frequency words. Furthermore, GPT is a generative pretraining model. Its feature extractor is composed of a multilayer transformer decoder. Compared to the fastText method, it can capture semantic information and recognize polysemy; therefore, the model using GPT as word embeddings is better than that using fastText in all aspects, with the F-score increased by 3%. However, although both BERT and GPT adopt transformers, BERT uses a bidirectional encoder. Thus, compared with the GPT model for capturing unidirectional information, BERT can use all context information and offers better advantages in word information extraction.

From the second experimental result, the precision rate, recall rate, and F-score of the three models, other than LSTM in the DDE’s relationship extraction task, were greater than 81%, implying that the application of the GRU method, which simplifies the LSTM model, is more feasible and effective. Compared with GRU, the F1 value of the BERT-GRU model was approximately 2% higher. This is because BERT can capture all context information and possesses a greater expressive ability to better complete information extraction tasks. Furthermore, compared with that of BERT-BiGRU, the F-score of BERT-BiGRU-ATT increased nearly by 4%. It is evident that increasing the attention mechanism can assign a higher weight to important information, which plays an important role in text feature extraction to further effectively complete the DDE’s relationship extraction task.

### 5.2. BERT-BiGRU-ATT Model Application

A few examples in the remaining data were randomly selected (after model training and testing) from 1927 users who asked questions more than five times in the 120ask dataset. The model was used to predict the relationship between entity pairs of Chinese Q&A data in OHC. The obtained results are presented in Table 5.

The relational classification model of BERT-BiGRU-ATT achieved better predictive effects with respect to relationship classification. Therefore, it can be used to extract DDE-related knowledge from OHCs.

**Figure 3:**
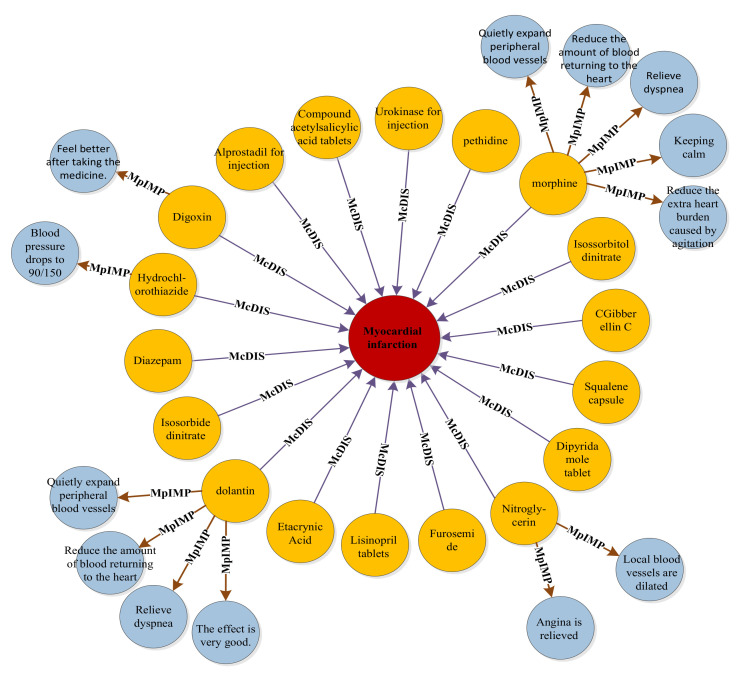
Drugs and drug effects related to myocardial infarction.

**Figure 4:**
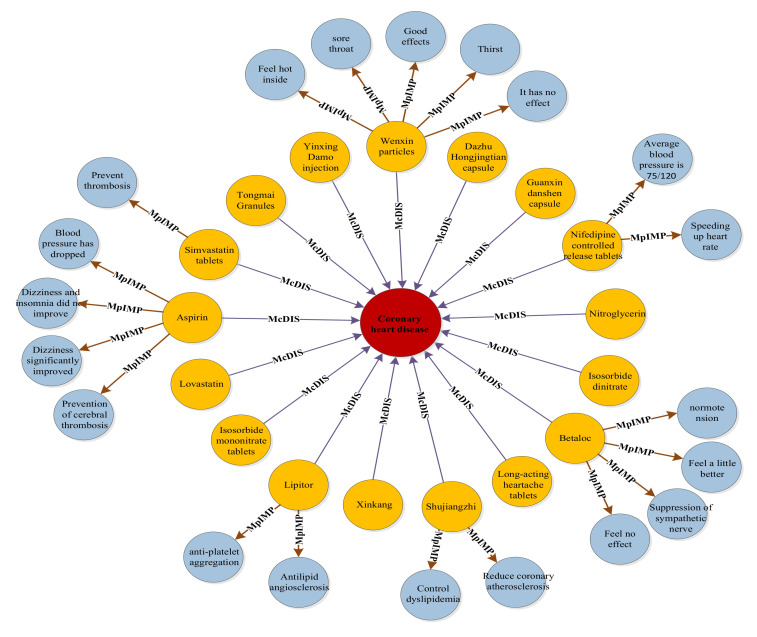
Drugs and drug effects related to coronary heart disease.

Considering “myocardial infarction” as an example, the final relation extraction results among the DDEs are shown in Figure 3. “Myocardial infarction” is associated with multiple drugs and drug effects. “Myocardial infarction,” drugs, and drug effects are represented by red, orange, and blue, respectively. The straight line represents the relationship between entities. Furthermore, considering “coronary heart disease” as an example, the relationship among DDEs is shown in Figure 4.

Life cycle health management refers to the user health management system for disease control and prevention. Health management involves formulating a personalized health management plan based on the individual’s health status, which can control and prevent diseases. Thus, this study identified the effective management of diseases, reduced medical expenses and medical accidents, and ultimately identified effective health care.

For the Q&A data in OHC, the DDEs of 1927 cardiovascular disease users were extracted according to the user ID, and the results were used to analyze the disease’s progression evolution from the first to the last question in three years. In addition, the results can be used to assist patient life cycle health management. Considering “Members 29231652” as an example, the user is a 27-year-old female and asked 14 questions during the three-year period. Based on the Q&A data and the relationship extraction results, the progression evolution of her hypertension medication was obtained, and the results are presented in Table 6.

## 6. Discussion and Conclusions

This paper proposed a BERT-GRU-ATT network architecture for the extraction of the relationship among DDEs from online health communities in China. The experimental results confirmed the effectiveness of the proposed model. BERT word embedding in the proposed model uses bidirectional encoding conversion in order to make better use of all important contextual information and grammatical and semantic feature information related to the biomedical field, and it has better advantages over Word2Vec, fastText, and GPT in word information extraction. Compared with LSTM, GRU, and BERT-BiGRU-ATT, the model of BERT-BiGRU-ATT showed better effects with respect to knowledge extraction, and it was superior in completing the knowledge extraction task of DDEs. This is due to the fact that BERT can capture all context information and has stronger expression abilities, and the execution of bidirectional GRU is also more efficient. In addition, the attention mechanism can assign higher weights to important information, which plays an important role in text feature extraction. Because the existing disease–drug relationship extraction is mostly based on electronic health records or abstracts from the medical literature, the greatest advantage of our research method is that information from unstructured, large-scale doctor–patient Q&A OHCs can be extracted. Furthermore, the knowledge extraction results can form a knowledge graph; according to the knowledge extraction results, the health management process of the dynamic life cycle of the medication process of a user’s disease is formed based on the user’s ID.

### 6.1. Theoretical Contributions

The theoretical contributions of our research are as follows: (1) Our research expands the research field on knowledge extraction. Currently, the research related to disease–drug knowledge extraction focuses on electronic medical records and medical literature summaries. Our study extends the research on disease–drug knowledge extraction to OHCs and expands the data sources of disease–drug research. (2) Our research expands the research direction of knowledge extraction from OHCs, enriches the knowledge sources of disease–drug management, and supplements and improves the existing knowledge system. (3) The disease–drug knowledge extraction method developed in our research serves as a reference for knowledge extraction in other fields.

### 6.2. Practical Implications

The practical implications of our research are as follows: (1) The results of knowledge extraction obtained in our research enrich the knowledge sources of disease medication and supplement and improve the existing knowledge base, which can assist doctors in clinical diagnosis and help patients in health management. (2) The extracted dynamic life-cycle disease evolution knowledge provides help for patients in conducting effective daily health management. Therefore, this study’s results have an important practical significance.

### 6.3. Limitations and Future Work

Although our research expands the research direction of knowledge extraction for disease medication management, there is still room for improvement, such as using coreference resolution, which can also be employed for DDE relationship extraction in future research to maximize the extraction of disease-related relationships in OHCs.

## Figures and Tables

**Table 1:** Annotation relationship statistics in corpus.

Dataset	DsDIS	DnsDIS	DpEFF	Others	Total
120ask	4246	1532	2461	1493	9732

**Table 2:** The performance of BERT-BiGRU-ATT on the test set.

Category	Precision	Recall	F-Score
DsDIS	89.04%	87.76%	88.40%
DnsDIS	86.85%	84.51%	85.67%
DpEFF	84.64%	85.67%	85.15%
Others	80.30%	78.37%	79.32%
Total	88.46%	86.09%	87.26%

**Table 3:** Comparison of the effects of different word embedding models.

Category	Precision	Recall	F-Score
Word2Vec-BiGRU-ATT (Baseline)	77.95%	78.53%	78.24%
fastText-BiGRU-ATT	79.45%	77.63%	78.53%
GPT-BiGRU-ATT	82.27%	80.76%	81.51%
BERT-BiGRU-ATT	88.46%	86.09%	87.26%

**Table 4:** Comparison of relationship classification results on test sets.

Category	Precision	Recall	F-Score
LSTM (Baseline)	72.15%	70.59%	71.36%
GRU	82.42%	81.06%	81.73%
BERT-BiGRU	84.95%	82.32%	83.61%
BERT-BiGRU-ATT	88.46%	86.09%	87.26%

**Table 5:** Prediction results on the Q&A data relationship extraction task.

Head Entity	Tail Entity	Q&A Sentences	True Relation	The Top Three Predication Relation (Probability)
arteriosclerosis	nifedipine sustained-release tablets	Hello, arteriosclerosis refers to many factors. Usually, you should pay attention to not smoking and not drinking alcohol, consuming nonhigh-fat, nonhigh-sugar, and nonhigh-salt foods, drinking plenty of water, and exercising properly. Suggestions: You can take some captopril, nifedipine sustained-release tablets or other drugs as appropriate and check your blood pressure regularly.	DsDIS	1. DsDIS (0.924124) 2. DpEFF (0.473218) 3. DnsDIS (0.145164)
antimalarials	favism	Favism is caused by mutations that affect the regulation of erythrocyte glucose-6-phosphate dehydrogenase; it is a hereditary hemolytic disease and is more common in men. Suggestions: It is necessary to pay attention to whether there is hemolysis; if there is, you need active treatment to prevent anemia and acute renal failure. Usually, you should avoid eating broad beans and their products, avoid taking drugs of oxidative properties (including antimalarials, sulfonamides, etc.), and actively prevent and treat it. The disease can still be controlled.	DnsDIS	1. DnsDIS (0.913346) 2. Others (0.336215) 3. DpEFF (0.074103)
captopril	blood pressure was still 100,160	It became apparent that I have had hypertension for more than two months, and my blood pressure was still 100,160 after taking captopril for one month. After that, I had taken hyzaar for a month, and my blood pressure dropped to 90,133. However, hyzaar is too expensive. Can I change to other less expensive drugs?	DpEFF	1. DpEFF (0.903671) 2. Others (0.384623) 3. DsDIS (0.104216)
Shensong Yangxin Capsule	myocardial ischemia	Can a 62-year-old man take a type of Shensong Yangxin Capsule for treating myocardial ischemia?	Others	1. Others (0.854143) 2. DsDIS (0.568127) 3. DnsDIS (0.134755)

Note: Each English relation instance is translated by a Chinese relation instance.

**Table 6:** Medication management information of a user’s DDEs and time evolution.

Time	Patient’s Question (Female, 27 Years Old, Hypertension)	Physician’s Reply
25 January 2018	“Nifedipine sustained-release tablets” have the effect of “accelerating heart rhythm.”	“Nifedipine sustained-release tablets” have the effect of “accelerating heart rhythm.”
25 January 2018	How should I take enalapril maleate tablet?	“Hypertension” applies to “enalapril maleate tablet,” and the specific dosage needs to be determined according to the individual’s constitution and condition.
26 January 2018	Drug effects of hypertension: higher low pressure, normal pressure high pressure.	“Hypertension” applies to “calcium antagonists” and “nifedipine sustained-release tablets.”
2 February 2018	Hypertensive drug effect: stable blood pressure.	Hypertension” applies to “amlodipine,” “irbesartan,” and “betaloc.”
8 February 2018	The effect of antihypertensive drugs: the amount of menstruation is light.	I suggest you evaluate further.
8 February 2018	“Nifedipine sustained-release tablets” has the drug effect of “increasing heart rate and uncomfortable heart;” “enalapril maleate tablet” has the effect of “not effective.”	“Hypertension” suits “nifedipine sustained-release tablets,” “benazepril tablets” and “betaloc” have the effect of “slow heart rate”
9 February 2018	“Enalapril maleate tablet” has the effect of being “not very effective.” Can I use hydrochlorothiazide tablets?	“Hypertension” can be treated with “enalapril maleate tablets” and “hydrochlorothiazide tablets;” “hydrochlorothiazide tablets” have a “hypokalemia” drug effect.
13 February 2018	I have hypertension, can I take enalapril maleate tablets, felodipine sustained-release tablets and betaloc simultaneously?	“Hypertension” can be treated with “enalapril maleate tablets,” “felodipine sustained-release tablets,” and “betaloc.”

Note: Each relationship instance is the translating from the Chinese characters.

## Data Availability

The data presented in this study are available upon request from the corresponding author. The data are not publicly available due to the inclusion of sensitive personal information.
